# TIC10/ONC201: a bend in the road to clinical development

**DOI:** 10.18632/oncoscience.133

**Published:** 2015-02-20

**Authors:** Yoshimi Endo Greer, Stanley Lipkowitz

**Affiliations:** Women's Malignancies Branch, Center for Cancer Research, National Cancer Institute, National Institutes of Health, Bethesda, Maryland

Tumor necrosis factor (TNF) –related apoptosis-inducing ligand (TRAIL) is an immuno-surveillance cytokine that is expressed in various tissues and cells [1, 2]. The expression of TRAIL on immune cells plays a role in immune surveillance in the prevention of tumors and metastasis [3]. TRAIL is attractive as an anti-tumor agent because of its capability to induce apoptosis in cancer cells by activating death receptors 4 and 5 (DR4 and DR5) with little toxicity against normal cells [2]. Despite its robust cell killing of tumor cells *in vitro,* efficacy of TRAIL receptor agonists has been limited in clinical trials, and this limited activity is thought to be due to lack of reliable predictive biomarkers of sensitivity and drug properties that limit efficacy such as short serum half-life, stability, and bio-distribution [3, 4].

**Figure 1:**
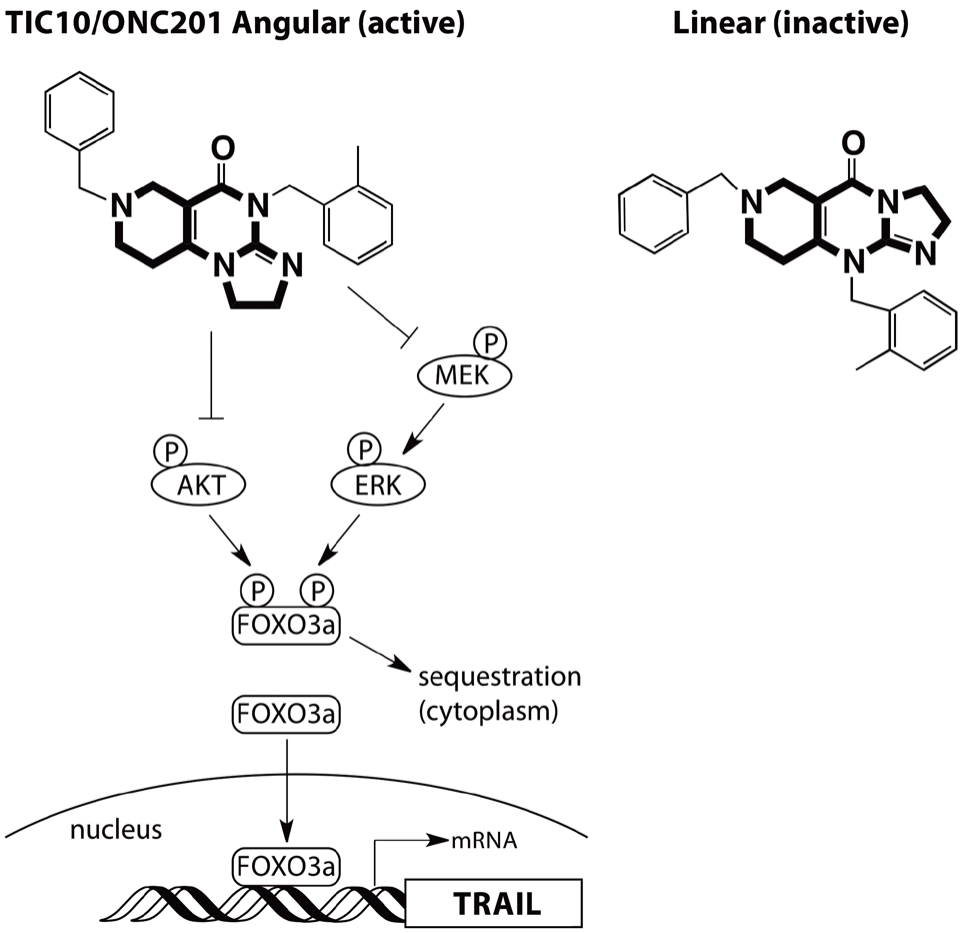
Structure and mechanism of action of TIC10/ONC201

To overcome the lack of activity of exogenous TRAIL ligands, Allen *et al.* conducted a screen using a colon cancer cell line to identify small molecules in a National Cancer Institute (NCI) chemical library that could induce TRAIL on tumor cells and thereby activate TRAIL receptors via an autocrine or paracrine mechanism [5]. This screen identified TRAIL-inducing compound 10 (TIC10; also known as ONC201 and NSC350625). TIC10/ONC201 induced sustained up-regulation of TRAIL in tumor cell lines and normal cells by dual-inhibition of Akt and MEK, leading to dephosphorylation of Foxo3a. This enables translocation of Foxo3a from the cytoplasm into the nucleus, where it binds to the TRAIL promoter to upregulate its gene transcription (Figure 1) [5]. TIC10/ONC201 induced TRAIL-mediated apoptosis in a wide range of cell lines and promoted tumor regression by inducing apoptosis in multiple xenograft tumor model systems including colon, breast and brain tumors [5]. With additional preferable features such as stability, activity by oral administration, and penetration of the blood brain barrier, TIC10/ONC201 is a novel anti-tumor therapeutic agent that acts on tumor cells and microenvironment by inducing TRAIL. This has led to planned clinical development of TIC10/ONC201 by Oncoceutics (source: http://oncoceutics.com/).

The isomeric structure of TIC10/ONC201 is critical to its activity [6, 7]. The structure was originally reported by Stähle *et al.* [8], and the molecule was described as an imidazolinopyrimidinone with a linear tricyclic core (Figure 1, inactive compound, tricyclic core highlighted in bold). Surprisingly, when Jacob *et al.* synthesized the reported structure to study its mechanism of action, it had no activity [6]. Subsequent NMR and X-ray crystallographic analyses of the original TIC10/ONC201 compound from the NCI library determined the active agent to be an isomer with an angular tricyclic core (Figure 1, active compound, tricyclic core highlighted in bold) [6]. Interestingly, a commercially available TIC10/ONC201 was found to be a third, inactive isomer [6]. The discrepancy between the reported structure and the active structure was confirmed by extensive analysis by Wagner *et al*. using NMR and X-ray crystallography [7]. Thus, close attention to the chemical structure of TIC10/ONC201 used in any further studies will be critical for preclinical and clinical development.

Despite the structural mis-assignment of TIC10/ONC201, the preclinical studies support the therapeutic potential of TIC10/ONC201. Elucidation of the structure of TIC10/ONC201 will facilitate its preclinical and clinical development. Indeed, the inactive isomers begin to provide structure/function insight. As this research moves forward, however, the challenge will be to address the hurdles that have hindered development of TRAIL receptor agonists. Predictive biomarkers that identify sensitive and/or resistant cells will need to be developed so that studies can be targeted to cohorts of patients most likely to benefit from TIC10/ONC201 [3]. In addition, a detailed understanding of mechanisms of resistance 1will be needed in each tumor type studied [3]. Finally, combination therapy that can overcome the drug resistance and/or lower the dose of drugs will be necessary. The structural bend in the road notwithstanding, TIC10/ONC201 is a promising and novel drug and should be developed further.
